# Intestinal Microbiota Community and Blood Fatty Acid Profiles of Albas Cashmere Goats Fed with Flaxseed Oil and Whole Flaxseed

**DOI:** 10.3390/ani13223531

**Published:** 2023-11-15

**Authors:** Yongmei Guo, Shulin Liu, Yinhao Li, Xiaoyu Guo, Yanli Zhao, Binlin Shi, Sumei Yan

**Affiliations:** Key Laboratory of Animal Nutrition and Feed Science at University of Inner Mongolia Autonomous Region, College of Animal Science, Inner Mongolia Agricultural University, Hohhot 010018, China; ymguo2015@163.com (Y.G.); liushulin0723@163.com (S.L.);

**Keywords:** grain, goat, different intestinal segments, bacteria, n-3 poly-unsaturated fatty acids

## Abstract

**Simple Summary:**

Manipulating dietary fatty acid composition is a feasible approach to improve the mutton fatty acid profiles of feedlot fattening goats. A previous study showed that flaxseed oil and grain affected the concentrations of c18:3n3, c20:5n3, c22:6n3, and n-3PUFA in the muscle tissues of Albas cashmere goats differently. The intestinal microflora may be involved in the regulation of blood lipid levels and their accumulation, as it plays an important role in the host’s metabolism. The specific results showed that the effects of flaxseed oil and grain on gut microbiota diversity vary in different segments in Albas cashmere goats. And flaxseed grain is more efficient than flaxseed oil in protecting intestinal health and promoting the absorption of c18:3n3.

**Abstract:**

The present study investigated the effects of flaxseed oil or flaxseed grain on the intestinal microbiota and blood fatty acid profiles of Albas cashmere goats. Sixty kid goats were allocated to three treatments and fed for 90 days with a control treatment, comprising a basal diet (CON, total-mixed ration with flaxseed meal), or experimental treatments, comprising a basal diet with added flaxseed oil (LNO) and a basal diet with added heated flaxseed grain (HLS). On day 90, two goats were randomly selected from each pen (eight goats per treatment) for euthanizing; then, five of the eight goats were randomly selected, and we collected their intestinal (duodenum, jejunum, ileum, cecum, and colon) digesta for analysis of the bacteria community. The results indicated that Firmicutes are the most predominant phylum in different segments of the intestinal digesta. Compared with the CON group, the relative abundance of duodenal Firmicutes, jejunal Saccharibacteria, and Verrucomicrobia significantly decreased in the LNO and HLS groups (*p* < 0.05), but there was no significant difference between the LNO and HLS groups. Compared with the CON and HLS groups, the RA of duodenal and jejunal Proteobacteria remarkably increased in the LNO group (*p* < 0.05), and there was no significant difference between the CON and HLS groups. Compared with the CON and LNO groups, the RA of Actinobacteria remarkably increased in the small intestine of the HLS group (*p* < 0.05), but there was no significant difference between the CON and LNO groups in the duodenum and ileum. The results of linear discriminant analysis (LDA) effect size (LEfSe) analysis showed that the HLS group was characterized by a higher RA of the [Eubacterium]_coprostanoligenes_group in the small intestine and the LNO group was represented by a higher RA of the Lachnospiraceae_NK3A20_group in the cecum and colon, while the CON group was represented by a higher RA of Solobacterium, Pseudoramibacter, and Acetitomaculum in the small intestine and a higher RA of norank_o__Bradymonadales, the Prevotellaceae_Ga6A1_group, and Ruminiclostridium_1 in the cecum and colon. In conclusion, the addition of flaxseed oil and grain rich in c18:3n3 to the diet could reduce the microbial diversity of the small intestinal segments and the microbial diversity and richness of the cecum and colon in Albas cashmere goats. And flaxseed grain is more efficient than flaxseed oil in protecting intestinal health and promoting the absorption of c18:3n3.

## 1. Introduction

Low levels of saturated fatty acids (SFAs) and high levels of n-3poly-unsaturated FAs (n-3PUFAs) in the diet are beneficial to human health [1,2]. An important source of n-3PUFAs is meat from ruminants. The Albas white cashmere goat is a world-renowned indigenous breed of cashmere- and meat-producing goat originating from the Ordos Plateau in Inner Mongolia, China. In recent years, the fattening model has shifted from traditional grazing to feedlot fattening as natural grazing resources have become limited, pastures have degenerated, and the demand for mutton has increased. However, feedlot fattening has changed the mutton fatty acid profiles, increased the SFA content, and decreased the n-3PUFA content in mutton [3]. Manipulating the dietary fatty acid composition might be a feasible approach to regulating cashmere goat meat fatty acid profiles. Our previous studies showed that the addition of flaxseed oil and flaxseed grain to the diet had different effects on increasing the concentrations of c18:3n3, c20:5n3, c22:6n3, and n-3PUFA in the muscle tissues of Albas cashmere goats, and flaxseed grain was more efficient [4]. 

The intestinal microbiota primarily influences the host by providing essential nutrients and non-nutrients, enhancing the host’s ability to obtain nutrients through the production of gastrointestinal enzymes, the modification of intestinal histology, and the creation of a physical barrier against pathogens [5,6]. Due to its important role in host metabolism, the gut microbiota may be involved in the regulation of blood lipid levels and lipid accumulation [7]. The fatty acids in animal blood mainly come from the absorption of the gastrointestinal tract, and the absorption of FAs in the gastrointestinal tract is affected by microbiota. In ruminants, current research is mainly focused on the effect of ruminal microbiota on PUFA hydrogenation [8,9], and there are few systematic reports exploring the impact of intestinal microbiota on PUFA absorption. The results of our previous study showed that, compared with a diet supplemented with linseed oil, flaxseed supplementation increased linoleic acid biohydrogenation by reducing the relative abundance of Ruminobacter and increasing the relative abundance of Prevotellace-ae_UCG-001 and Fretibacterium in the rumen, protecting c18:3n3 from biohydrogenation and resulted in higher levels of n-3-PUFAs in the post-intestinal tract [10], and both linseed oil and flaxseed grain significantly increased the content of c18:3n3, c20:5n3, and n-3PUFA in blood. Flaxseed grain was more efficient than linseed oil; flaxseed grain promoted c22:6n3 but linseed oil did not [4]. The previous results showed that dietary supplementation with flaxseed oil or grain changed the ileal microbiota composition; flaxseed grain ameliorated the blood lipid profiles, but flaxseed oil did not. The changes in the blood lipid profiles of cashmere goats in response to dietary supplementation with flaxseed grain were probably related to the change in the ileal microbiota composition [11]. Therefore, we hypothesize that the effects of flaxseed oil or grain on the blood lipid profiles of Albas cashmere goats are related to changes in the diversity of the intestinal microbiota, and further affect host health through a variety of crosstalk mechanisms.

In view of this, the effects of flaxseed oil and flaxseed supplementation on the intestinal microbiota of Albas cashmere goats should be investigated, and studies should further explore whether the shift in the blood fatty acid profiles of cashmere goats in response to dietary supplementation with flaxseed oil or grain is related to the change in intestinal microbiota composition and explain the possible mechanisms by which flaxseed oil and grain ameliorate blood fatty acid profiles. 

This study was conducted at the Inner Mongolia White Cashmere Goat Breeding Farm, Wulan Town, Etuoke Banner, Ordos City, Inner Mongolia Autonomous Region, China (39°12′ N; 107°97′ E). All animal procedures were performed in accordance with the National Standard Guidelines for Ethical Review of Animal Welfare (GB/T 35892-2018) [12].

### 1.1. Experimental Design, Diet, and Feeding Management

Sixty 4-month-old, castrated Albas white cashmere male kid goats (average body weight 18.6 ± 0.1 kg) were selected and randomly divided into three treatments, with each treatment comprising four pens of five kid goats. The control treatment group (CON) was fed the total-mixed ration (TMR) with no supplementation. The experimental treatment group was fed the TMR with added flaxseed oil (LNO). In the second experimental treatment group, heated flaxseed grain (HLS; flaxseed contains about 36% oil and was roasted for 10 min at 120 °C) was added to the TMR, with the same flaxseed oil content as in the LNO treatment. The diet was formulated according to the nutritional requirements for meat goats [13] (Table 1), and the dietary nutrition level was adjusted in the different fattening stages. All kid goats were adapted to the TMR diet for 14 days; the experimental period was 90 days, including early (1 to 30 days), medium (31 to 60 days), and late (61 to 90 days) fattening stages. The daily rations were divided into two portions: one portion was offered at 08:30 and the other was offered at 16:30. All animals always had access to fresh water. 

### 1.2. Sample Collection

On day 90, two goats were randomly selected from each pen (eight goats per group) for euthanizing, and then five of the eight goats were randomly selected for microbial community analysis. Before slaughter, the goats fasted for 24 h and were prohibited from drinking water for 2 h. After slaughter, the intestine was opened and the duodenum, large intestine, and colon were separated using a suture line to avoid reflux between adjacent areas of the gastrointestinal tract. The digesta samples were collected separately and homogenized. Finally, homogenized samples from each segment of the digestive tract were frozen in liquid nitrogen and then stored at −80 °C for analysis of microbial composition.

### 1.3. DNA Extraction and Checking

Seventy-five digesta samples were thawed at 4 °C and kept on ice throughout the extraction process. Microbial DNA was extracted using the E.Z.N.A.^®^ Soil DNA kit (Omega Bio-tek, Norcross, GA, USA) in accordance with the instructions for use. Final DNA concentration and OD (optical density) 260/280 nm were measured using a Nanodrop 2000 UV-vis (ultraviolet-visible) spectrophotometer (Thermo Scientific, Wil-mington, NC, USA), and DNA quality was verified by 1% agarose gel electrophoresis.

### 1.4. PCR Amplification and Checking

The V3-V4 regions of the bacterial 16S rRNA genes were amplified in triplicate using PCR (95 °C for 3 min of denaturation, followed by 27 cycles at 95 °C for 30 s, 55 °C for 30 s of annealing, 72 °C for 45 s of elongation, and a final extension at 72 °C for 10 min). The universal primers used in this amplification protocol were: 338F (5′-ACCHOCTACGGGAGGCAGCAG-3′) and 806R (5′-GGACTACHVGGGTWCHOTAAT-3′). The PCR reactions were conducted in a 20 μL mixture containing: 4 μL of 5 × FastPfu Buffer, 2 μL of 2.5 mM dNTPs (Deoxynucleotide Triphosphates), 0.8 μL of each primer (5 μM), 0.4 μL of FastPfu Polymerase, and 10 ng of template DNA. The PCR products were excised from a 2% agarose gel. Concerning purification, an AxyPrep DNA Gel Extraction Kit (Axygen Biosciences, Union City, CA, USA) was used and quantified using QuantiFluor™-ST (Promega, Madison, WI, USA) according to the instruction manual.

### 1.5. Illumina MiSeq Sequencing

Purified amplicons were pooled in equimolar and paired-end sequenced (2 × 300 bp) on an Illumina MiSeq PE300 instrument (Illumina, San Diego, CA, USA) according to the standard protocols of Majorbio Bio-Pharm Technology Co. Ltd. (Shanghai, China).

### 1.6. Bioinformatics

Raw reads of each sample were demultiplexed and quality filtered using default parameters in Quantitative Insights into Microbial Ecology through QIIME (Quantitative Insights into Microbial Ecology, version 1.9.1) software, quality filtered using Trimmomatic, and merged using FLASH (Fast Length Adjustment of Short Reads). Low-quality reads were removed according to the following criteria: (i) The reads were truncated at any site receiving an average quality score <20 over a 50 bp sliding window and truncated reads shorter than 50 bp were discarded; (ii) reads with 2 nucleotide mismatches in primer matching or that contained ambiguous characters were removed; (iii) sequences that overlapped by at least 10 bp were assembled based on their overlap sequences. The assembled sequences were assigned to operational taxonomic units (OTUs), which are the most used microbial diversity units, were clustered with a 97% similarity cutoff using UPARSE (Highly Accurate OTU Sequences from Microbial Amplicon Reads, version 7.1, http://drive5.com/uparse/, accessed on 1 October 2013), and chimeric sequences were identified and removed using UCHIME (Chimera Prediction for Amplicon Sequencing). Alpha (Coverage, Sobs, Ace, Chao, Shannon, and Simpson) and beta diversities were calculated for downstream analysis of OTUs. OTUs were taxonomically analyzed using the Ribosomal Database Project (RDP) Classifier algorithm (http://rdp.cme.msu.edu/, accessed on 30 September 2016). A rarefaction curves’ analysis with Mothur v.1.21.1 was performed to reflect the sequence depth. Principal coordinate analysis (PCoA) was applied to visualize the dissimilarity of microbial communities among different groups using the weighted Unifrac distance with R Language.

### 1.7. Statistical Analysis

The bacterial diversity indexes and the relative abundance of bacteria at the phylum level were analyzed in SAS (SAS Inst. Inc., Cary, NC, USA). The data were checked for normality of variance. The data obeying normal distribution were still analyzed using a one-way analysis of variance (ANOVA), and Duncan’s multiple range tests were carried out. The data that disobeyed normal distribution were analyzed using the Kruskal–Wallis test. The results were presented as the mean values and standard error of the mean (SEM). Data means significance was declared at *p* ≤ 0.05 and tendencies were considered at 0.05 < *p* ≤ 0.10; there was no significance at *p* > 0.10. The differences in microbial community abundance at the genus level between the groups, and the effects of each differentially abundant taxon, were assessed using the non-parametric factorial Kruskal–Wallis sum-rank test and the linear discriminant analysis (LDA) effect size (LEfSe) method, which emphasized statistical significance and biological correlation. The threshold was set at an LDA level of > 2, *p* < 0.05. Spearman correlation was used to correlate the blood fatty acid profiles with the differential bacterial genera through CCREPE software (version 1.7.0); the data for the blood fatty acid profiles were obtained from our previous results [4]. Correlations with |R| > 0.5 and *p* ≤ 0.05 for the linear model were considered as significant.

## 2. Results

### 2.1. Sequencing Data 

**Figure 1:**
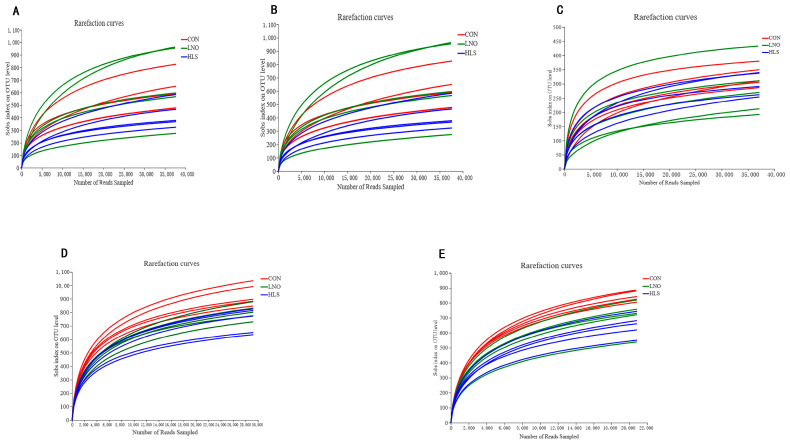
The OTU rarefaction curves of the intestinal digesta bacterial communities. Curves were drawn using the least-sequenced sample as the upper limit for the rarefactions. Each color represents one treatment: the red curves represent kids fed the basal diet (CON), the green curves represent kids fed the basal diet supplemented with flaxseed oil (LNO), and the blue curves represent kids fed the basal diet supplemented with heated flaxseed grain (HLS). (**A**): duodenum; (**B**): jejunum; (**C**): ileum; (**D**): cecum; (**E**): colon.

In the present study, fifteen digesta samples were collected and analyzed for three different treatments in each region of the intestine. To facilitate unified analysis, the results of the 15 samples from the three different treatments were normalized according to the minimum sequence in one sample. A total of 4,111,507 optimized sequences were obtained after quality filtering with an average of (54,820 ± 6500) optimized sequences per sample (Table 2). As shown in Figure 1, the individual-based rarefaction curves generated for each sample were sufficient to accurately describe the bacterial composition of intestinal digesta. The results showed that the sampling depth was adequate to estimate bacterial community.

### 2.2. The Effect of Flaxseed Oil and Grain on the Bacterial Community in Different Intestinal Tracts

#### 2.2.1. Intestinal Bacterial α- and β-Diversity

**Figure 2:**
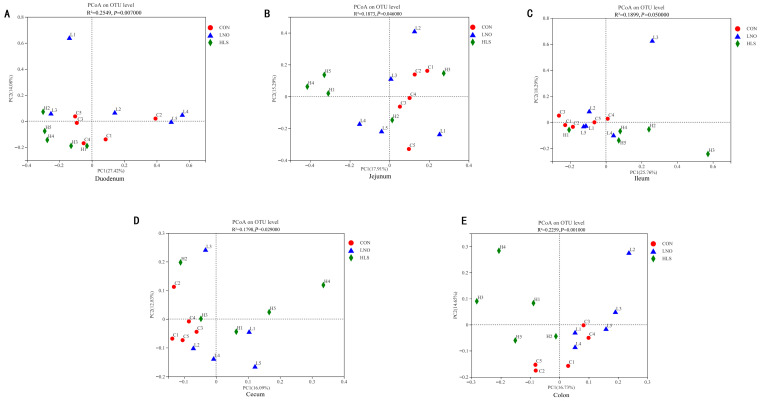
Principal coordinate analysis (PCoA, using the weighted Unifrac similarity metric) of bacterial operational taxonomic units (OTUs) in the intestinal digesta of kid goats. Each symbol represents one treatment: The solid red circle represents kids fed with the basal diet (CON), the solid blue triangle represents kids fed with the basal diet supplemented with flaxseed oil (LNO), and the solid green rhombus represents kids fed with the basal diet supplemented with heated flaxseed grain (HLS). (**A**): duodenum; (**B**): jejunum; (**C**): ileum; (**D**): cecum; (**E**): colon.

As indicated in Table 3, the coverage (the coverage estimator) index of the intestinal digesta in all groups was 0.991–0.999, which indicated that the sequencing data were sufficiently representative. In the duodenum and ileum, compared with the LNO group, the Sobs (the richness estimator), Ace (the richness estimator), and Chao (the richness estimator) indexes significantly increased in the CON and HLS groups (*p* < 0.05), but there was no significant difference between CON and HLS groups. In the jejunum, compared with the HLS group, the Sobs, Shannon, Ace, and Chao indexes significantly increased in the CON and LNO groups (*p* < 0.05), but the Simpson (the diversity estimator) index remarkably decreased, and there was no significant difference between the CON and LNO groups. In the cecum and colon, compared with the CON group, the Sobs, Shannon, Ace, and Chao indexes significantly decreased in the HLS and LNO groups (*p* < 0.05), but the Simpson index remarkably increased, and there was no significant difference between the HLS and LNO groups. At the OTU level, the principal coordinate analysis (PCoA) plots (Figure 2) demonstrate dissimilarities between the CON group, the LNO group, and the HLS group (*p* ≤ 0.05).

#### 2.2.2. Bacterial Composition at the Phylum Level

**Figure 3:**
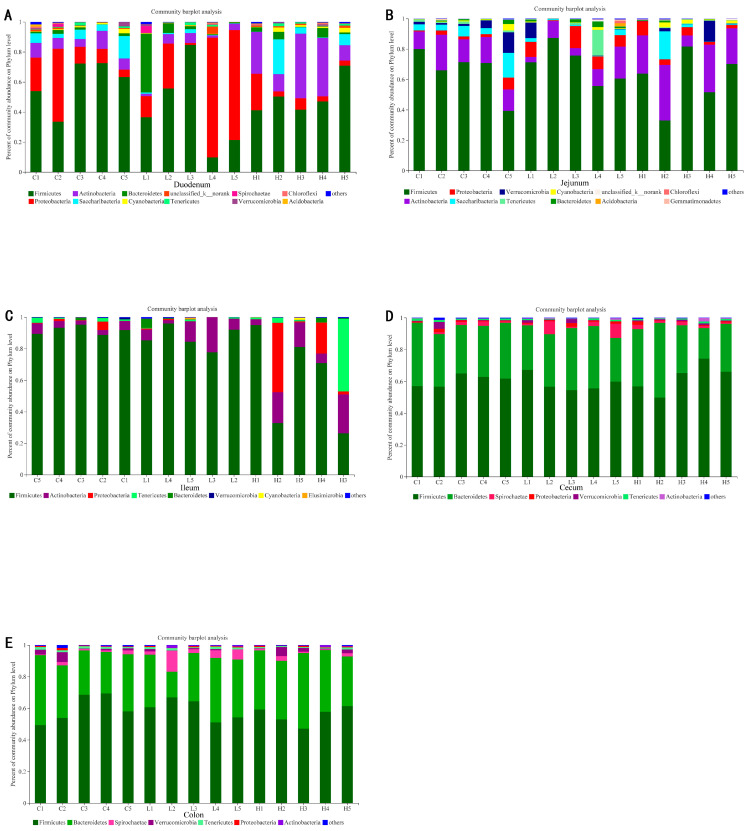
The bar chart shows the phylum-level bacterial composition of intestinal digesta. The x-coordinate represents the samples; the Y-coordinate indicates the relative abundance of bacteria at the phylum level. (**A**): duodenum; (**B**): jejunum; (**C**): ileum; (**D**): cecum; (**E**): colon.

As shown in Figure 3, a detailed overview of the bacterial composition of the intestinal digesta in each sample was illustrated at the phylum level. The Firmicutes are the most predominant phylum in different segments of intestinal digesta. As shown in Table 4, compared with the CON group, the relative abundance (RA) of duodenal Firmicutes, jejunal Saccharibacteria, and Verrucomicrobia decreased in the LNO and HLS groups (*p* < 0.05), but there was no significant difference between the LNO and HLS groups. Compared with the CON and HLS groups, the RA of duodenal and jejunal Proteobacteria and colonic Spirochaetae increased in the LNO group (*p* < 0.05); the duodenal Saccharibacteria’s RA showed the opposite result (*p* < 0.05), and there was no significant difference between the CON and HLS groups. Compared with the CON and LNO groups, the RA of duodenal Actinobacteria and Bacteroidetes, ileal Actinobacteria, and cecal Verrucomicrobia increased in the HLS group (*p* < 0.05); the RA of ileal Firmicutes, Proteobacteria, Tenericutes and colonic Tenericutes showed the opposite result (*p* < 0.05), but there was no significant difference between the CON and LNO groups. In the jejunum, the RAs of Actinobacteria in the LNO group, CON group, and HLS group were increased (*p* < 0.0001). In the cecum, the Firmicutes’ RA in the HLS group is remarkably higher than in the LNO group (*p* = 0.027), but the CON group did not differ from the LNO and HLS groups. In the colon, the RAs of Firmicutes in the LNO group, HLS group, and CON group were decreased (*p* = 0.009).

#### 2.2.3. Bacterial Composition at Genus Level 

**Figure 4:**
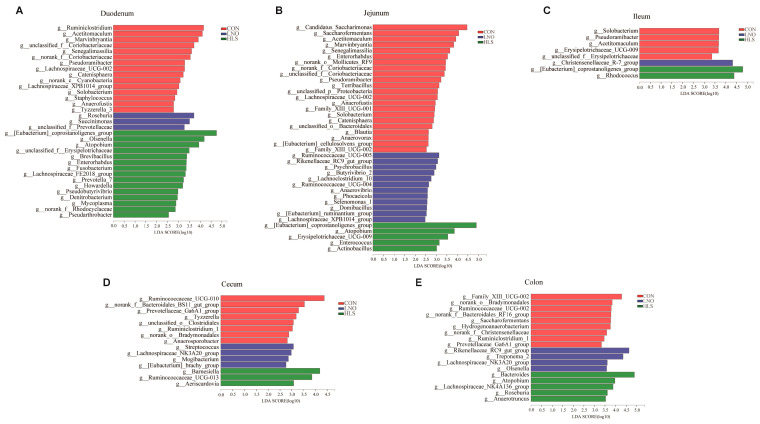
Linear discriminant analysis (LDA) identified distinct bacterial genera that were enriched in the CON, LNO, and HLS groups. Genera with LDA score > 2 and *p* < 0.05 were considered significant. Each color represents one treatment: the red represents kids fed the basal diet (CON), the blue represents kids fed the basal diet supplemented with flaxseed oil (LNO), and the green represents kids fed the basal diet supplemented with heated flaxseed grain (HLS). (**A**): duodenum; (**B**): jejunum; (**C**): ileum; (**D**): cecum; (**E**): colon.

LEfSe (LDA > 2) was used to investigate the microbial genus that the differences between the LNO, HLS, and CON groups. As shown in Figure 4, differential expression analysis showed that the RAs of multiple genera in the duodenum, jejunum, ileum, cecum, and colon were significantly different between the LNO, HLS, and CON groups. In the duodenum (Figure 4A), 15 genera were significantly enriched in the HLS group compared to the CON and LNO groups: Enterorhabdus, the [Eubacterium]_coprostanoligenes_group, Fusobacterium, Olsenella, Brevibacillus, Atopobium, Howardella, Pseudobutyrivibrio, the Lachnospiraceae_FE2018_group, Denitrobacterium, norank_f__Rhodocyclaceae, Mycoplasma, Pseudarthrobacter, Prevotella_7, unclassified_f__Erysipelotrichaceae; three genera were remarkably enriched in the LNO compared to the CON and HLS groups: Roseburia, Succinimonas, unclassified_f__Prevotellaceae; and 15 genera were remarkably enriched in the CON compared to the LNO and HLS groups: the Lachnospiraceae_XPB1014_group, Catenisphaera, unclassified_f__Coriobacteriaceae, Anaerofustis, Marvinbryantia, Solobacterium, norank_c__Cyanobacteria, Senegalimassilia, Lachnospiraceae_UCG-002, Pseudoramibacter, Ruminiclostridium, Acetitomaculum, norank_f__Coriobacteriaceae, Staphylococcus, Tyzzerella_3. 

In the jejunum, five genera were more abundant in the HLS group than in the LNO and CON groups: the [Eubacterium]_coprostanoligenes_group, Actinobacillus, Atopobium, Enterococcus, Erysipelotrichaceae_UCG-009; 12 genera were more abundant in the LNO group than in the HLS and CON groups: the Lachnospiraceae_XPB1014_group, Selenomonas_1, Ruminococcaceae_UCG-005, Ruminococcaceae_UCG-004, Phocaeicola, Butyrivibrio_2, Domibacillus, the [Eubacterium]_ruminantium_group, Lachnoclostridium_10, the Rikenellaceae_RC9_gut_group, Anaerovibrio, Psychrobacillus; and 22 genera were more abundant in the CON group than in the HLS and LNO groups: Enterorhabdus, unclassified_o__Bacteroidales, unclassified_p__Proteobacteria, unclassified_f__Coriobacteriaceae, Anaerofustis, Marvinbryantia, Solobacterium, Anaerovorax, Candidatus_Saccharimonas, Saccharofermentans, Family_XIII_UCG-002, Family_XIII_UCG-001Blautia, Senegalimassilia, Pseudoramibacter, Catenisphaera, Acetitomaculum, Terribacillus, norank_f__Coriobacteriaceae, the [Eubacterium]_cellulosolvens_group, norank_o__Mollicutes_RF9, Lachnospiraceae_UCG-002 (Figure 4B).

In the ileum, two genera were significantly enriched in the HLS group compared to the CON and LNO groups: the [Eubacterium]_coprostanoligenes_group, Rhodococcus; one genera was remarkably enriched in the LNO compared to CON and HLS groups: the Christensenellaceae_R-7_group; and five genera were remarkably enriched in the CON compared to the LNO and HLS groups: Pseudoramibacter, unclassified_f__Erysipelotrichaceae, Solobacterium, Acetitomaculum, Erysipelotrichaceae_UCG-009 (Figure 4C). 

In the cecum, three genera were more abundant in the HLS group than in the LNO and CON groups: Aeriscardovia, Barnesiella, Ruminococcaceae_UCG-013; four genera were more abundant in the LNO group than in the HLS and CON groups: the Lachnospiraceae_NK3A20_group, Streptococcus, Mogibacterium, the [Eubacterium]_brachy_group; and eight genera were more abundant in the CON group than in the HLS and LNO groups: the Prevotellaceae_Ga6A1_group, Ruminiclostridium_1, Tyzzerella, Anaerosporobacter, the norank_f__Bacteroidales_BS11_gut_group, norank_o__Bradymonadales, unclassified_o__Clostridiales, Ruminococcaceae_UCG-010 (Figure 4D).

In the colon, five genera were more abundant in the HLS group than in the LNO and CON groups: Roseburia, Atopobium, Bacteroides, Anaerotruncus, the Lachnospiraceae_NK4A136_group; four genera were more abundant in the LNO group than in the HLS and CON groups: the Lachnospiraceae_NK3A20_group, Rikenellaceae_RC9_gut_group, Olsenella, Treponema_2; and nine genera were more abundant in the CON group than in the HLS and LNO groups: Hydrogenoanaerobacterium, norank_f__Christensenellaceae, the Prevotellaceae_Ga6A1_group, Ruminiclostridium_1, Family_XIII_UCG-002, Ruminococcaceae_UCG-002, norank_o__Bradymonadales, Saccharofermentans, the norank_f__Bacteroidales_RF16_group (Figure 4E).

**Figure 5:**
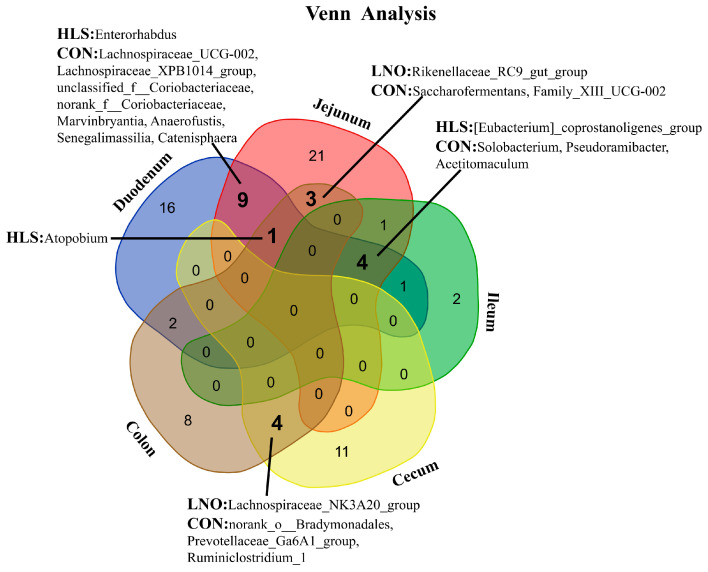
Venn diagram analysis of shared distinct bacteria at genus level in the intestinal digesta of kid goat. Each color represents one site: the blue represents the duodenum, the red represents the jejunum, the green represents the ileum, the yellow represents the cecum, and the brown represents the colon.

To further identify the shared genera in different intestinal segments, as shown in Figure 5, we found one shared genus ([Eubacterium]_coprostanoligenes_group) in the digesta samples of the duodenum, jejunum, and ileum, one shared genus (Atopobium) in the digesta samples of the duodenum, jejunum, and colon, and one shared genus (Enterorhabdus) in the digesta samples of the duodenum and jejunum, which were significantly increased in the HLS group compared to the CON and LNO groups. Of these, one shared genus (Rikenellaceae_RC9_gut_group) in the digesta samples of the jejunum and colon, and one shared genus (Lachnospiraceae_NK3A20_group) in the digesta samples of the cecum and colon, were significantly increased in the LNO group compared to the CON and HLS groups. Of these, three shared genera (Solobacterium, Pseudoramibacter, Acetitomaculum) in the digesta samples of the duodenum, jejunum and ileum, eight shared genera (Lachnospiraceae_UCG-002, the Lachnospiraceae_XPB1014_group, unclassified_f__Coriobacteriaceae, norank_f__Coriobacteriaceae, Marvinbryantia, Anaerofustis, Senegalimassilia, Catenisphaera) in the digesta samples of the duodenum and jejunum, two shared genera (Saccharofermentans, Family_XIII_UCG-002) in the digesta samples of the jejunum and colon, and three shared genera (norank_o__Bradymonadales, the Prevotellaceae_Ga6A1_group, Ruminiclostridium_1) in the digesta samples of the cecum and colon were significantly increased in the CON compared to the HLS and LNO groups.

### 2.3. Spearman’s Correlation Analysis

Spearman correlation analysis was conducted between the differential bacterial genera in different intestinal tracts and blood fatty acid profiles, as indicated in Table 5, Table 6, Table 7, Table 8 and Table 9. The data set of the blood fatty acid profiles was obtained from our previous research [4] (Table S1). The threshold |R| > 0.5 and *p* ≤ 0.05 is considered as a significant Spearman correlation.

In the duodenum (Table 5), the [Eubacterium]_coprostanoligenes_group was positively associated with c18:3n3 and c20:5n3, but was negatively correlated with c20:4n6, Anaerofustis, Marvinbryantia, Senegalimassilia, and Solobacterium showed the opposite effect. Acetitomaculum, Denitrobacterium, Lachnospiraceae_UCG-002, and Pseudoramibacter were negatively correlated with c18:3n3 and c20:5n3. The [Eubacterium]_coprostanoligenes_group and unclassified_f__Erysipelotrichaceae were negatively correlated with c16:0. Brevibacillus was negatively correlated with c18:0. Marvinbryantia, Pseudarthrobacter, and Pseudoramibacter were positively associated with c18:1c9, but the [Eubacterium]_coprostanoligenes_group was negatively correlated with it. Pseudarthrobacter was negatively correlated with c18:2c6. norank_f__Coriobacteriaceae and unclassified_f__Coriobacteriaceae were negatively correlated with C18:3n3. The Lachnospiraceae_XPB1014_group was positively associated with c20:4n6, but Olsenella was negatively correlated with it. Enterorhabdus was negatively correlated with c20:5n3.

In the jejunum (Table 6), the [Eubacterium]_coprostanoligenes_group was positively associated with C18:3n3, but was negatively correlated with c20:4n6; Family_XIII_UCG-001 and norank_o__Mollicutes_RF9 showed the opposite effect. Anaerofustis, Catenisphaera, Lachnospiraceae_UCG-002, Pseudoramibacter, Saccharofermentans, Solobacterium, and unclassified_f__Coriobacteriaceae were negatively correlated with c18:3n3 and c20:5n3 but were positively associated with c20:4n6. The [Eubacterium]_cellulosolvens_group was positively associated with c18:1c9 but was negatively correlated with c20:5n3. Norank_f__Coriobacteriaceae was positively associated with c16:0 but was negatively correlated with C20:5n3. Anaerofustis and Catenisphaera were positively associated with c18_1c9. Marvinbryantia and Senegalimassilia were negatively correlated with c18:3n3. Blautia was positively associated with c20:4n6, but Enterococcus was negatively correlated with it.

In ileum (Table 7), unclassified_f__Erysipelotrichaceae, Solobacterium, Erysipelotrichaceae_UCG-009, and Acetitomaculum were negatively correlated with c18:3n3 and c20:5n3, but were positively associated with c20:4n6; the [Eubacterium]_coprostanoligenes_group showed the opposite effect. Solobacterium and Erysipelotrichaceae_UCG-009 were positively associated with c16:0. The [Eubacterium]_coprostanoligenes_group was positively associated with c22:6n3, but Christensenellaceae_R-7_group was negatively correlated with it.

In the cecum (Table 8), Ruminiclostridium_1 was negatively correlated with c18:3n3 and c20:5n3. Ruminococcaceae_UCG-010 was positively associated with c18:1c9. Prevotellaceae_Ga6A1_group was positively associated with c20:4n6, but was negatively correlated with c18:3n3 and c20:5n3. Barnesiella was positively associated with c18:0. Norank_o__Bradymonadales was negatively correlated with c20:5n3. The norank_f__Bacteroidales_BS11_gut_group was positively associated with c20:4n6. The [Eubacterium]_brachy_group and Mogibacterium were positively associated with c20:5n3.

In the colon (Table 9), Roseburia, Bacteroides, and Anaerotruncus were positively associated with c22:6n3. Ruminococcaceae_UCG-002 was positively associated with c18:2c6 and c20:4n6. Ruminiclostridium_1, norank_f__Bacteroidales_RF16_group, Prevotellaceae_Ga6A1_group, Saccharofermentans, norank_f__Christensenellaceae, Family_XIII_UCG-002, Hydrogenoanaerobacterium, and norank_o__Bradymonadales were negatively correlated with c18:3n3 and c20:5n3; except for norank_f__Christensenellaceae and norank_o__Bradymonadales, the other bacteria were positively correlated with c20:4n6.

### 2.4. Bacterial Community in Different Intestinal Tracts Independent of the Diet

**Figure 6:**
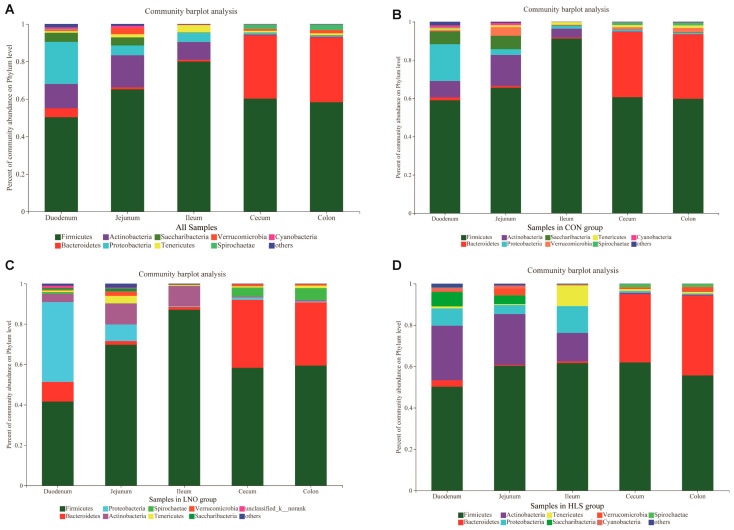
Average relative abundance of the dominant phyla (phyla with average relative abundance ≥ 0.01 in at least one region) in 5 intestinal regions of cashmere goats. The X-coordinate represents the samples, the Y-coordinate indicates the relative abundance of bacteria at the phylum level. (**A**): All groups; (**B**): CON group; (**C**): LNO group; (**D**): HLS group).

At the phylum level, 50 bacteria phyla were detected in the intestinal digesta of the Albas cashmere goats. The number of bacterial phyla detected in the duodenum, jejunum, ileum, cecum, and colon of CON, LNO, and HLS goats were 39, 30, 33; 28, 34, 33; 18, 18, 21; 18, 18, 16; 15, 14, 16, respectively. As indicated in Figure 6, the small intestinal (duodenum, jejunum, and ileum) digesta were dominated by Firmicutes (50.30–79.98%), Actinobacteria (9.47–17.11%), and Proteobacteria (5.22–22.37%). The large intestinal digesta (cecum and colon) were dominated by Firmicutes (58.27–60.26%) and Bacteroidetes (33.62–34.50%). 

**Figure 7:**
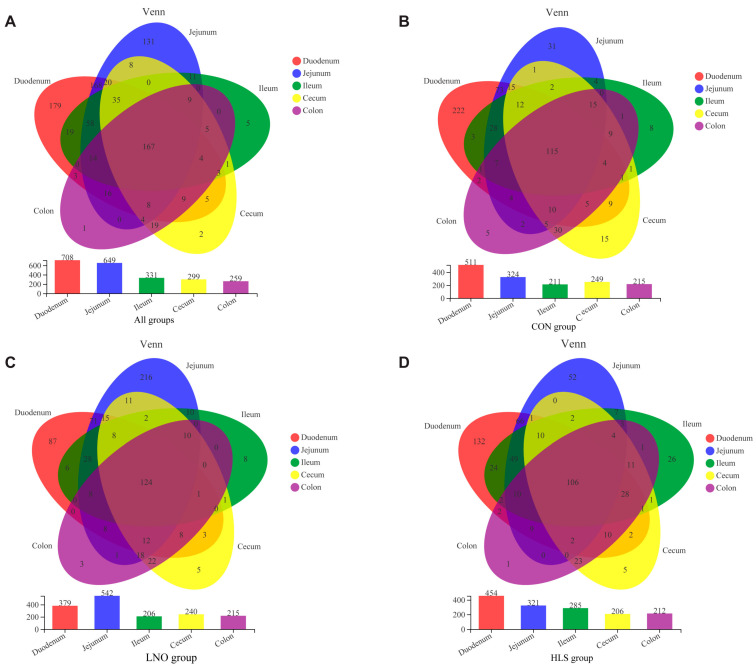
Venn diagram analysis of generic level bacterial composition in the intestinal digesta of goat kids. (**A**): All groups (n = 75). (**B**): CON group. (**C**): LNO group. (**D**): HLS group. Each color represents one site: the red represents duodenum, the blue represents jejunum, the green represents ileum, the yellow represents cecum, and the purple represents colon. The bar chart shows the number of genera in each segment.

At the genus level, a Venn daigram was used to evaluate the common and exclusive bacterial genus in the intestinal digesta of each group. The data indicated that 708, 649, 331, 299, and 259 genera were found in the digesta of the duodenum, jejunum, ileum, cecum, and colon, respectively, and 167 genera were found to be shared among the different intestinal tracts independent of the diet (Figure 7A). The data indicated that 511, 324, 211, 249, and 215 genera were found in the digesta of the duodenum, jejunum, ileum, cecum, and colon, respectively, and 115 genera were found to be shared among the different intestinal tracts in the CON group (Figure 7B). The data indicated that 379, 542, 206, 240, and 215 genera were found in the digesta of the duodenum, jejunum, ileum, cecum, and colon, respectively, and 124 genera were found to be shared among the different intestinal tracts in the LNO group (Figure 7C). The data indicated that 454, 321, 285, 206, and 212 genera were found in the digesta of the duodenum, jejunum, ileum, cecum, and colon, respectively, and 106 genera were found to be shared among the different intestinal tracts in the HLS group (Figure 7D). 

**Figure 8:**
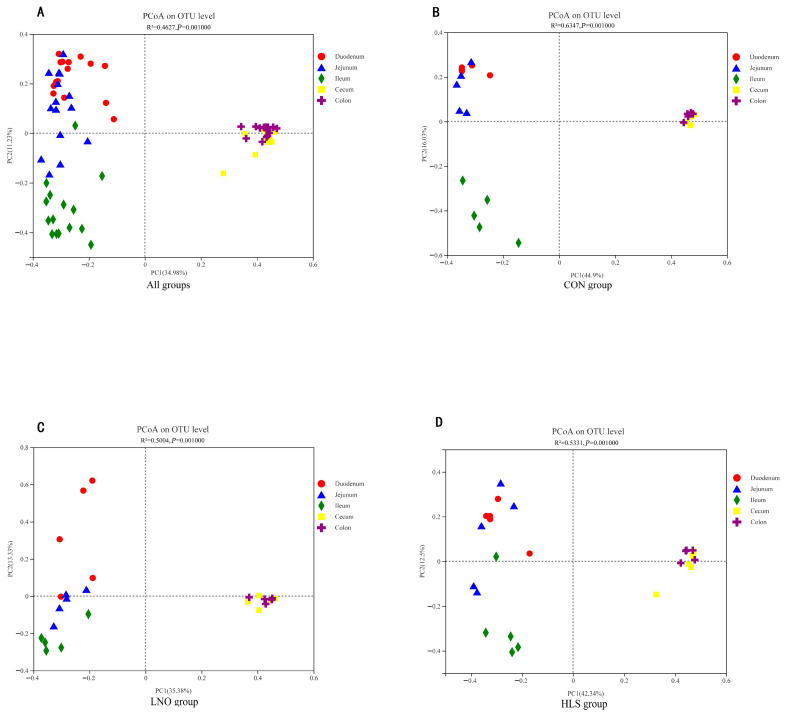
Principal coordinate analysis (PCoA, using the weighted Unifrac similarity metric) of bacterial operational taxonomic units in 5 intestinal segments of Albas cashmere goats. (**A**) All groups (n = 75). (**B**): CON group. (**C**): LNO group. (**D**): HLS group. Each symbol represents one site: the solid red circle represents duodenum, the solid blue triangle represents jejunum, the solid green rhombus represents ileum, the solid yellow square represents cecum, and the solid purple cross-shaped represents colon.

At the OTU level, PCoA was used based on the Bray–Curtis dissimilarity matrices to estimate the bacterial community structure of each group in the intestine; the figure shows that the bacterial communities of the small intestine (duodenum, jejunum, and ileum) and large intestine (cecum and colon) were very different from each other (Figure 8A–D). Small intestine samples (duodenum, jejunum, and ileum) occupied the left side of PC1, while large intestine samples (cecum and colon) occupied the right side of PC1.

## 3. Discussion

This study provided a detailed picture of bacterial community dynamics along the intestinal tract segments in cashmere goats, and the relationship between the significantly differential genera and blood FA profiles. The bacterial community richness was measured using the Sobs, Chao, and Ace indexes, and the diversity was measured using the Shannon and Simpson indexes [13]. In the present study, compared with the CON group, the results showed that the dietary flaxseed oil significantly decreased the richness and diversity of the duodenal bacterial community and the diversity of the ileal bacterial community; the addition of flaxseed grain notably decreased the richness and diversity of the jejunal bacterial community. The supplementation of flaxseed oil and grain significantly decreased the richness and diversity of bacteria in the cecum and colon of cashmere goats. Huyben et al. (2020) [14] reported that fish fed with a high lipid diet with high n-3 PUFA had reduced alpha-diversity in the gut microbiome [15], Cremonesi et al. (2018) reported that linseed supplementation affected dairy goats’ ruminal bacteria population, with a significant reduction in biodiversity [16], and these results were similar to our results. This can be explained by the fact that FAs may have the function of lysis and solubilization of bacterial cell membranes [17,18], or of being used as metabolic substrates by intestinal bacteria, thus influencing the profile of the intestinal microbiota [19].

The intestinal microflora is a major regulator of host metabolism. The composition and function of the gut microbiota are dynamic and are influenced by diet, such as by the quantity and profile of fatty acids. Consequently, dietary fatty acids can influence host physiology by interacting with the gut microbiota. The n-3 PUFAs are mainly absorbed in the gut, where some microorganisms can directly utilize n-3 PUFAs and produce numerous small molecules [7]. Dietary PUFAs play a crucial role in the structure of the microbial community in the host. Adding fish oil (rich in n-3PUFA) to the low-fat diet of mice C57BL/6J significantly decreased the RA of Firmicutes, and decreased weight gain and inflammatory bowel diseases [20]. The Verrucomicrobia phylum, which colonizes the intestinal tract of humans and animals, is phylogenetically related to Planctomycetes and Chlamydiae and consists mainly of Akkermansia species [21]. The gram-negative bacterium Akkermansia muciniphila, which has been isolated from human feces, can degrade the mucosa of the intestinal epithelium [21], and the pili-like structural protein of these bacteria may be directly involved in regulating intestinal immunity and enhancing resistance to transport across the intestinal epithelium [22]. In this study, compared with the CON group, the flaxseed oil and grain significantly decreased the RA of duodenal Firmicutes and jejunal Verrucomicrobia, which indicates that the flaxseed oil and grain were beneficial to intestinal health. Proteobacteria is a major phylum of gram-negative bacteria with a wide range of pathogenic microorganisms [23], and a bloom of Proteobacteria in the gut reflects dysbiosis or an unstable gut microbial community structure [24]. Stecher (2013) reported that human intestinal pathogens belong to a small group of bacterial families belonging to the Proteobacteria, proteobacterial “blooms” being a feature of an abnormal microbiota, for example following antibiotic therapy, dietary change, or inflammation [25]. In the present study, compared with the CON and HLS groups, the flaxseed oil significantly increased the RA of Proteobacteria, which indicated that the flaxseed oil may increase the risk of pathogenic invasion and affect the absorption of c18:3n3. Actinobacteria is the most dominant phylum in the bacteria domain and is a prolific source of new bioactive compounds [26]. Actinobacteria produce a variety of bioactive compounds of medical importance, including antibacterial, antifungal, antiviral, anticancer, and neuroprotective compounds [27,28,29]. By 2010, at least 34,000 bioactive compounds had been detected in bacteria, and 40% of them were produced by Actinobacteria [30]. In the present study, compared with the CON and LNO groups, the dietary flaxseed grain supplementation significantly increased the RA of Actinobacteria in the small intestine. The results indicated that flaxseed grain can promote the beneficial bacteria Actinobacteria colonized in the small intestine, protect intestinal mucosal epithelial cells from injury, and then promote the absorption of c18:3n3 and n-3PUFA into the blood. Taken as a whole, dietary flaxseed grain increased the RA of probiotics, but the flaxseed oil increased the individual harmful bacteria; these results indicated that the flaxseed grain was more efficient than flaxseed oil in protecting the health of the intestinal tract and helping more c18:3n3 be absorbed into the blood, thereby increasing the content of c18:3n3 in the blood, which further explained the results of the previous study [4]. 

Acetomaculum is mainly found in ruminants fed with a concentrate-rich diet and can use monosaccharides to produce acetate [31]. Pseudoramibacter is one of the bacteria which promote the generation of short chain fatty acids (SCFAs) [32]. Solobacter is a gram-positive and anaerobic bacterium, and the products of glucose fermentation are mainly acetic acid, lactic acid, and butyric acid, with lesser amounts of pyruvic acid [33]. In the present study, compared with the CON group, the flaxseed oil and grain significantly decreased the RAs of Acetitomaculum, Solobacterium and Pseudoramibacter, which indicates that flaxseed oil and grain decreased the content of organic acids in the intestines, but high concentration of organic acids induce apoptosis of intestinal epithelial cells [34], so flaxseed oil and grain can protect the intestine from injury, and promote the absorption of c18:3n3. The results of the correlation analysis in the present study showed that duodenal Solobacterium, Acetitomaculum, and Pseudoramibacter were negatively correlated with c18:3n3, jejunal Solobacterium and Pseudoramibacter were negatively correlated with c18:3n3, and ileal Solobacterium and Acetitomaculum were negatively correlated with c18:3n3. It was further confirmed that the above three bacteria were not conducive to the absorption of c18:3n3 in the small intestine. Hu et al. (2019) reported that the RA of the Prevotellaceae Ga6A1 group was significantly increased in diabetic mice, which was positively correlated with pyruvic acid [35]. Liu et al. (2020) reported that Ruminiclostridium can produce butyric acid, and liraglutide significantly decreased the RA of Ruminiclostridium in the intestine of mice with nonalcoholic fatty liver disease, which was positively correlated with alanine aminotransferase and aspartate aminotransferase [36]. In the present study, compared with the CON group, flaxseed oil and grain significantly reduced the RA of the Prevotellaceae Ga6A1 group and Ruminiclostridium_1 in the cecum and colon, which indicated that the flaxseed oil and grain contributed to the proliferation and repair of intestinal epithelial cells, improved intestinal integrity and barrier function, and improved the digestive and barrier protection function of the intestine. The results of the correlation analysis showed that the Prevotellaceae Ga6A1 group and Ruminiclostridium_1 were negatively correlated with the c18:3n3, further indicating that the Prevotellaceae Ga6A1 group and Ruminiclostridium_1 were detrimental to absorption of c18:3n3. The [Eubacterium]_coprostanoligenes_group is one of the probiotics generating beneficial SCFAs, which have an anti-inflammatory effect [37]. Sphingosine produced by the [Eubacterium]_coprostanoligenes_group could enter the circulator stream, further regulate the host lipid homeostasis, and alleviate dyslipidemia [38]. In the present study, in the small intestine, the RA of the [Eubacterium]_coprostanoligenes_group was significantly higher in the HLS group than in the CON and LNO groups, which illustrated that flaxseed grain could increase the RA of probiotics and protect the intestinal epithelium from damage, and further absorb more c18:3n3 into the blood, thereby increasing the content of c18:3n3 in the blood [4] and alleviating the dyslipidemia [11]. The correlation analysis showed that the [Eubacterium]_coprostanoligenes_group was positive with c18:3n3, further confirming the above results. 

At the phylum level, independent of diet (that is, in the same treatment), Firmicutes were the most abundant bacteria in the intestine of cashmere goats, and the Firmicutes, Actinobacteria, and Proteobacteria were the predominant bacterial phyla in the small intestine; the Firmicutes and Bacteroides were the predominant bacterial phyla in cecum and colon (Figure 5). The number of the major and minor phyla of bacteria indicated that the large intestine may have lower diversity but a higher abundance of bacterial phyla, and the small intestine is occupied by numerous minor phyla that may participate in a higher number of processes [39]. The results of PCoA and Adonis analysis (Figure 7) showed that the bacteria differed distinctly among the small intestine and large intestine. Zhang et al. (2020) reported the microbial community in the small intestine was different from the cecum sample of Yak [40], which was similar to our results. In essence, the composition of the intestinal microbiota was consistent in cashmere goats fed with different diets, which was consistent with Li’s report, but the relative abundance of microbiota fluctuated with changes in diet [13]. The present data indicated that the dominant bacteria in the same intestinal tract of goats from different treatments were consistent, but the relative abundance was changed, which may be related to the change in diet. Therefore, we investigated the effects of different dietary supplementations on intestinal bacteria in the same intestinal tract. 

Feedlot fattening compromised the mutton quality, which could not meet the consumers’ demand for high-quality meat products. Based on the results of this study and previous research by our group, adding flaxseed grain to the diet of cashmere goats under feedlot fattening would be an efficient way to protect the intestinal health of cashmere goats and improve the quality of mutton by enrichment of c18:3n3 in muscle and fat tissue.

## 4. Conclusions

The addition of flaxseed oil and grain rich in c18:3n3 to the diet could reduce the microbial diversity of the small intestinal segments, and the microbial diversity and richness of the cecum and colon of cashmere goats. A reduction in the RA of the duodenal Firmicutes phylum, the jejunal Verrucomicrobia phylum, and the harmful bacteria Solobacterium, Acetitomaculum, and Pseudoramibacter in the small intestine, plus the decrease in the RA of Ruminiclostridium_1 and the Prevotellaceae_ Ga6A1_group in the cecum and colon, implied that flaxseed oil and grain could effectively protect intestinal health and promote the absorption of c18:3n3 and n-3PUFA into blood. Compared to flaxseed oil, flaxseed grain increased the RA of the probiotic Actinobacteria phylum and the [Eubacterium]_coprostanoligenes_group in the small intestine of cashmere goats, as the grain is more helpful for the absorption of c18:3n3.

## Figures and Tables

**Table 1:** The composition and nutrient levels of CON, LNO, and HLS groups (dry-matter basis, DM basis).

Ingredients	Day 1 to 30			Day 31 to 60			Day 61 to 90		
	CON	LNO	HLS	CON	LNO	HLS	CON	LNO	HLS
Alfalfa	25.00	25.00	25.00	15.00	15.00	15.00	12.50	12.50	12.50
Corn stalk	5.00	5.00	5.00	20.00	20.00	20.00	25.00	25.00	25.00
Oat	20.00	20.00	20.00	15.00	15.00	15.00	12.50	12.50	12.50
Corn	28.41	23.37	23.17	30.80	30.40	29.90	31.30	29.90	29.40
Soybean meal 46%	11.70	10.50	11.50	9.50	11.40	11.90	8.00	10.40	10.90
Distiller’s dried grains with soluble, DDGS	3.00	7.24	7.74	4.00	0.50	0.50	4.00	0.50	0.50
Flaxseed meal	4.80	4.80	0.00	3.50	3.50	0.00	4.50	4.50	0.00
Flaxseed	0.00	0.00	5.50	0.00	0.00	5.50	0.00	0.00	7.00
Flaxseed oil	0.00	2.00	0.00	0.00	2.00	0.00	0.00	2.50	0.00
Premix (1)	0.50	0.50	0.50	0.50	0.50	0.50	0.50	0.50	0.50
Limestone	0.20	0.20	0.20	0.20	0.20	0.20	0.20	0.20	0.20
CaHPO4	0.20	0.20	0.20	0.20	0.20	0.20	0.20	0.20	0.20
NaCl	0.54	0.54	0.54	0.50	0.50	0.50	0.50	0.50	0.50
NaHCO3	0.35	0.35	0.35	0.80	0.80	0.80	0.80	0.80	0.80
MgO	0.30	0.30	0.30	0.00	0.00	0.00	0.00	0.00	0.00
Total	100.00	100.00	100.00	100.00	100.00	100.00	100.00	100.00	100.00
Nutrient levels									
Digestible energy, DE MJ/Kg DM (2)	12.83	13.09	13.06	12.87	13.00	12.96	12.74	13.09	13.05
Dry Matter, DM/%	88.02	88.36	88.24	89.14	89.36	89.32	87.09	87.02	87.03
Crude protein, CP g/kg DM	188.73	188.13	188.20	162.84	158.71	159.69	153.52	151.34	151.85
Ether extract, EE g/kg DM	29.12	53.97	53.97	28.99	45.84	46.14	26.85	48.99	49.92
Neutral Detergent Fiber, NDF g/kg DM	425.31	431.18	441.60	448.60	427.41	439.12	457.42	436.06	450.68
Acid Detergent Fiber, ADF g/kg DM	232.20	237.71	243.3	242.59	235.23	248.40	247.69	240.32	256.96
Calcium, Ca g/kg DM	11.25	11.11	11.00	10.48	10.89	10.78	10.26	10.67	10.56
Phosphorus, P g/kg DM	4.65	4.67	4.78	4.50	4.44	4.56	4.31	4.22	4.33

(1) Each kilogram of premix provided the following: iron (Fe) 4 g, copper (Cu) 0.8 g, zinc (Zn) 5 g, manganese (Mn) 3 g, iodine (I) 30 mg, selenium (Se) 30 mg, cobalt (Co) 25 mg, vitamin (VA) 600,000 IU (International Unit), vitamin D (VD3) 250,000 IU, vitamin E (VE) 1250 IU, vitamin K (VK3) 180 mg, vitamin B1 (VB1) 35 mg, vitamin B2 (VB2) 850 mg, vitamin B6 (VB6) 90 mg, nicotinic acid 2200 mg, D-pantothenic acid 1700 mg, vitamin B12 (VB12) 3 mg, biotin 14 mg, folic acid 150 mg. (2) Digestible energy was calculated based on the ingredients of the diet and their digestible energy content and not based on the actual dry matter intake. CON: basal diet; LNO: basal diet added with flaxseed oil; HLS: basal diet with heated flaxseed grain.

**Table 2:** Optimized sequencing data of intestinal digesta.

Group	Optimized Sequences	Average Sequences	Mean_Base	Mean_Length	Min_Length	Max_Length
Duodenum						
CON	224,418	44,884	18,521,788	412.57	247	480
LNO	244,950	48,990	20,493,991	417.72	245	480
HLS	282,979	56,596	23,178,454	409.65	247	474
Jejunum						
CON	309,498	61,900	25,363,009	409.88	246	446
LNO	310,366	62,073	25,579,668	412.12	230	450
HLS	274,223	54,845	22,469,544	409.65	243	448
Ileum						
CON	271,857	54,371	23,336,091	429.08	291	467
LNO	281,019	56,204	24,221,281	431.32	280	481
HLS	253,906	50,781	21,967,437	432.89	283	474
Cecum						
CON	313,157	62,631	25,849,679	412.71	263	457
LNO	245,830	49,166	20,323,122	413.30	236	449
HLS	319,688	63,938	26,381,252	412.50	236	456
Colon						
CON	297,818	59,564	24,586,815	412.72	275	454
LNO	216,507	43,301	17,895,233	413.11	276	439
HLS	265,291	53,058	21,920,157	413.16	238	463

CON: basal diet; LNO: basal diet with added flaxseed oil; HLS: basal diet with added heated flaxseed grain.

**Table 3:** Effects of dietary flaxseed oil or heated flaxseed grain on bacterial α-diversity index of intestinal digesta.

Items	CON	LNO	HLS	SEM	*p*-Value
Duodenum					
Sobs	717A	462B	626A	47.33	0.008
Shannon	3.81	2.92	3.90	0.27	0.230
Simpson	0.08	0.15	0.07	0.04	0.990
Ace	812.91A	611.61B	779.29A	43.19	0.014
Chao	865.17A	557.30B	791.07A	58.45	0.008
Coverage	0.997	0.997	0.997	0.0005	0.988
Jejunum					
Sobs	689A	772A	384B	55.24	0.0008
Shannon	4.07A	4.08A	3.30B	0.11	0.0003
Simpson	0.04B	0.05B	0.12A	0.01	<0.0001
Ace	860.16A	905.30A	485.12B	56.34	0.0003
Chao	839.03A	901.77A	505.29B	47.21	0.0001
Coverage	0.996	0.996	0.997	0.0005	0.312
Ileum					
Sobs	325A	253B	355A	13	0.038
Shannon	2.69	2.56	2.64	0.19	0.931
Simpson	0.16	0.14	0.17	0.03	0.748
Ace	418.86A	356.34B	404.77A	3.71	0.001
Chao	407.55A	354.18B	408.35A	5.75	0.019
Coverage	0.999	0.999	0.999	0.0002	0.722
Cecum					
Sobs	931A	803B	740B	34.52	0.006
Shannon	5.33A	4.95B	4.81B	0.07	0.001
Simpson	0.01B	0.02A	0.02A	0.002	0.014
Ace	1062.50A	908.79B	860.71B	39.92	0.01
Chao	1095.83A	919.42B	867.17B	38.27	0.003
Coverage	0.994	0.994	0.995	0.0004	0.329
Colon					
Sobs	847A	713B	654B	29.05	0.002
Shannon	5.28A	4.90B	4.74B	0.12	0.019
Simpson	0.013B	0.025A	0.023A	0.002	0.004
Ace	989.43A	841.48B	793.75B	34	0.004
Chao	992.68A	857.26B	800.52B	35.43	0.007
Coverage	0.991	0.992	0.992	0.0004	0.149

A, B: Means within the same row not followed by the same letters are significantly different at *p* ≤ 0.05, whereas the differences were considered to be a statistical trend when 0.05 < *p* ≤ 0.10. CON: basal diet; LNO: basal diet added with flaxseed oil; HLS: basal diet added with heated flaxseed grain. SEM: standard error of the mean. Sobs: the number of OTUs; ACE: the ACE estimator; Chao: the Chao estimator.

**Table 4:** Effects of dietary flaxseed oil or heated flaxseed grain on bacterial composition of intestinal digesta (phylum level).

Phylum, %	CON	LNO	HLS	SEM	*p*-Value
Duodenum					
p__Firmicutes	65.43A	37.73B	45.02B	3.83	0.001
p__Proteobacteria	12.00B	49.28A	4.41B	7.4	0.003
p__Actinobacteria	8.35B	4.08B	26.28A	3.99	0.005
p__Saccharibacteria	4.90A	0.55B	5.80A	0.5	0.008
p__Bacteroidetes	1.75B	1.07B	3.83A	0.56	0.046
Jejunum					
p__Firmicutes	64.27	70.06	60.08	7.41	0.644
p__Actinobacteria	14.42B	9.07C	29.08A	1.55	<0.0001
p__Proteobacteria	2.24B	8.63A	3.26B	0.42	0.006
p__Saccharibacteria	4.67A	0.56B	0.89B	0.37	0.008
p__Verrucomicrobia	1.40A	0.27B	0.23B	0.06	0.005
Ileum					
p_Firmicutes	90.80A	87.22A	75.83B	1.826	0.015
p_Actinobacteria	4.96B	8.92B	20.06A	1.449	0.001
p_Proteobacteria	0.79A	0.71A	0.37B	0.106	0.042
p_Tenericutes	1.25A	1.03A	0.62B	0.102	0.005
p_Bacteroidetes	1.48B	1.49B	2.78A	0.641	0.040
Cecum					
p__Firmicutes	60.74AB	56.56B	62.76A	1.41	0.027
p__Bacteroidetes	34.02	33.39	31.99	1.83	0.732
p__Spirochaetae	1.37	1.54	1.65	0.21	0.657
p__Proteobacteria	0.64	0.83	0.55	0.11	0.160
p__Verrucomicrobia	0.40B	0.30B	0.66A	0.06	0.004
Colon					
p__Firmicutes	53.81C	64.08A	59.54B	0.99	<0.0001
p__Bacteroidetes	30.94	33.36	36.17	1.41	0.067
p__Spirochaetae	1.37B	4.00A	1.49B	0.56	0.01
p__Verrucomicrobia	1.16	0.67	1.38	0.3	0.271
p__Tenericutes	1.38A	1.40A	0.63B	0.06	0.009

A, B, C: Means within the same row not followed by the same letters are significantly different at *p* ≤ 0.05, whereas the differences were considered to be a statistical trend when 0.05 < *p* ≤ 0.10. CON: basal diet; LNO: basal diet with added flaxseed oil; HLS: basal diet with added heated flaxseed grain. SEM: standard error of the mean.

**Table 5:** Correlation analysis of differential duodenal bacteria and blood fatty acid composition.

Genus	C16_0		C18_0		C18_1c9		C18_2c6		C18_3n3		C20_4n6		C20_5n3		C22_6n3	
	*p*-Value (1)	R (2)	*p*-Value	R	*p*-Value	R	*p*-Value	R	*p*-Value	R	*p*-Value	R	*p*-Value	R	*p*-Value	R
g__[Eubacterium]_coprostanoligenes_group	0.033	−0.543			0.0002	−0.630			<0.0001	0.865	0.043	−0.548	<0.0001	0.720		
g__Acetitomaculum									0.014	−0.541			<0.0001	−0.618		
g__Anaerofustis									0.001	−0.698	0.010	0.627	0.004	−0.609		
g__Brevibacillus			0.012	−0.560												
g__Denitrobacterium									0.044	−0.504			0.012	−0.568		
g__Enterorhabdus													0.000	−0.549		
g__Lachnospiraceae_UCG-002									0.003	−0.673			0.0003	−0.712		
g__Lachnospiraceae_XPB1014_group											0.010	0.516				
g__Marvinbryantia					0.008	0.725			0.0001	−0.792	0.014	0.635	<0.0001	−0.772		
g__norank_f__Coriobacteriaceae									0.046	−0.530						
g__Olsenella											0.001	−0.562				
g__Pseudarthrobacter					0.050	0.556	0.041	−0.526								
g__Pseudoramibacter					0.021	0.761			0.001	−0.738			0.008	−0.736		
g__Senegalimassilia									0.004	−0.627	0.014	0.567	0.0002	−0.640		
g__Solobacterium									0.002	−0.581	0.023	0.507	0.000	−0.659		
g__unclassified_f__Coriobacteriaceae									0.012	−0.534						
g__unclassified_f__Erysipelotrichaceae	0.005	−0.557														

(1) Correlations with *p* ≤ 0.05 for the linear model were considered as significant. (2) Spearman correlation coefficient (R) represents the degree of association between differential bacteria and fatty acid profiles in blood, spearman’s correlation analysis with an R value > 0.5 indicates a positive correlation and a value <−0.5 indicates negative correlation.

**Table 6:** Correlation analysis of differential jejunal bacteria and blood fatty acid composition.

Genus	C16_0		C18_0		C18_1c9		C18_2c6		C18_3n3		C20_4n6		C20_5n3		C22_6n3	
	*p*-Value (1)	R (2)	*p*-Value	R	*p*-Value	R	*p*-Value	R	*p*-Value	R	*p*-Value	R	*p*-Value	R	*p*-Value	R
g__[Eubacterium]_cellulosolvens_group					0.006	0.566							0.04	−0.579		
g__[Eubacterium]_coprostanoligenes_group									<0.0001	0.718	<0.0001	−0.776				
g__Anaerofustis					0.005	0.699			<0.0001	−0.751	0.023	0.556	0.0004	−0.749		
g__Blautia											0.002	0.586				
g__Catenisphaera					0.046	0.557			0.0002	−0.753	0.0005	0.626	<0.0001	−0.779		
g__Enterococcus											0.002	−0.58				
g__Family_XIII_UCG-001									0.002	−0.601	0.004	0.527				
g__Lachnospiraceae_UCG- 002									0.002	−0.685	0.037	0.612	0.03	−0.534		
g__Marvinbryantia									0.011	−0.52						
g__norank_f__Coriobacteriaceae	0.027	0.514											0.002	−0.541		
g__norank_o__Mollicutes_RF9									0.008	−0.569	0.001	0.597				
g__Pseudoramibacter									0.008	−0.67	0.034	0.567	0.0001	−0.723		
g__Saccharofermentans									0.0002	−0.653	0.044	0.586	0.007	−0.581		
g__Senegalimassilia									0.019	−0.519						
g__Solobacterium									0.001	−0.714	0.027	0.573	0.009	−0.682		
g__unclassified_f__Coriobacteriaceae									<0.0001	−0.732	0.021	0.682	0.002	−0.548		

(1) Correlations with *p* ≤ 0.05 for the linear model were considered as significant. (2) Spearman correlation coefficient (R) represents the degree of association between differential bacteria and fatty acid profiles in blood, spearman’s correlation analysis with an R value > 0.5 indicates a positive correlation and a value <−0.5 indicates negative correlation.

**Table 7:** Correlation analysis of differential ileal bacteria and blood fatty acid composition.

Genus	C16_0		C18_0		C18_1c9		C18_2c6		C18_3n3		C20_4n6		C20_5n3		C22_6n3	
	*p*-Value (1)	R (2)	*p*-Value	R	*p*-Value	R	*p*-Value	R	*p*-Value	R	*p*-Value	R	*p*-Value	R	*p*-Value	R
g__unclassified_f__Erysipelotrichaceae									0.003	−0.598	0	0.631	0.007	−0.543		
g__Solobacterium	0.014	0.658							0	−0.729	0	0.718	0.003	−0.843		
g__[Eubacterium]_coprostanoligenes_group									0.001	0.799	0.001	−0.863	0.022	0.62	0.032	0.585
g__Erysipelotrichaceae_UCG-009	0.001	0.724							0.001	−0.694	0.001	0.752	0.001	−0.782		
g__Acetitomaculum									0.002	−0.851	0.003	0.839	0.028	−0.705		
g__Christensenellaceae_R−7_group															0.032	−0.625

(1) Correlations with *p* ≤ 0.05 for the linear model were considered as significant. (2) Spearman correlation coefficient (R) represents the degree of association between differential bacteria and fatty acid profiles in blood, spearman’s correlation analysis with an R value > 0.5 indicates a positive correlation and a value <−0.5 indicates negative correlation.

**Table 8:** Correlation analysis of differential cecal bacteria and blood fatty acid composition.

Genus	C16_0		C18_0		C18_1c9		C18_2c6		C18_3n3		C20_4n6		C20_5n3		C22_6n3	
	*p*-Value (1)	R (2)	*p*-Value	R	*p*-Value	R	*p*-Value	R	*p*-Value	R	*p*-Value	R	*p*-Value	R	*p*-Value	R
g__Ruminiclostridium_1									0.020	−0.540			0.000	−0.574		
g__Ruminococcaceae_UCG-010					0.004	0.716										
g__Prevotellaceae_Ga6A1_group									0.012	−0.625	0.001	0.582	0.003	−0.634		
g__Barnesiella			0.026	0.561												
g__norank_o__Bradymonadales													0.042	−0.508		
g__norank_f__Bacteroidales_BS11_gut_group											0.009	0.621				
g__[Eubacterium]_brachy_group													0.013	0.551		
g__Mogibacterium													0.003	0.586		

(1) Correlations with *p* ≤ 0.05 for the linear model were considered as significant. (2) Spearman correlation coefficient (R) represents the degree of association between differential bacteria and fatty acid profiles in blood, spearman’s correlation analysis with an R value > 0.5 indicates a positive correlation and a value <−0.5 indicates negative correlation.

**Table 9:** Correlation analysis of differential colonic bacteria and blood fatty acid composition.

Genus	C16_0		C18_0		C18_1c9		C18_2c6		C18_3n3		C20_4n6		C20_5n3		C22_6n3	
	*p*-Value (1)	R (2)	*p*-Value	R	*p*-Value	R	*p*-Value	R	*p*-Value	R	*p*-Value	R	*p*-Value	R	*p*-Value	R
g__Roseburia															0.002	0.588
g__Bacteroides															0.004	0.751
g__Anaerotruncus															0.005	0.558
g__Ruminococcaceae_UCG-002							0.006	0.553			0.0001	0.658				
g__Ruminiclostridium_1									0.0003	−0.642	<0.0001	0.614	0.003	−0.644		
g__norank_f__Bacteroidales_RF16_group									0.006	−0.652	0.0002	0.682	0.003	−0.586		
g__Prevotellaceae_Ga6A1_group									0.001	−0.763	0.0001	0.661	0.001	−0.732		
g__Saccharofermentans									0.004	−0.553	0	0.514	0.006	−0.54		
g__norank_f__Christensenellaceae									0.006	−0.509			0.006	−0.596		
g__Family_XIII_UCG-002									0.008	−0.619	0.0005	0.636	0.0001	−0.527		
g__Hydrogenoanaerobacterium									<0.0001	−0.68	0	0.712	0.005	−0.551		
g__norank_o__Bradymonadales									0.042	−0.52			0.004	−0.562		

(1) Correlations with *p* ≤ 0.05 for the linear model were considered as significant. (2) Spearman correlation coefficient (R) represents the degree of association between differential bacteria and fatty acid profiles in blood, spearman’s correlation analysis with an R value > 0.5 indicates a positive correlation and a value <−0.5 indicates negative correlation.

## Data Availability

The sequencing data are available from the National Center for Biotechnology Information under the Sequence Read Archive (SRA) with the BioProject No. PRJNA809885, PRJNA810318, PRJNA810285, PRJNA810293, PRJNA810302.

## Supplementary Materials

The following supporting information can be downloaded at: https://www.mdpi.com/article/10.3390/ani13223531/s1, Table S1: Fatty acid profiles in plasma of kid goats.
